# Twenty-Five Years of Domoic Acid Monitoring in Galicia (NW Spain): Spatial, Temporal, and Interspecific Variations

**DOI:** 10.3390/toxins13110756

**Published:** 2021-10-25

**Authors:** Juan Blanco, Ángeles Moroño, Fabiola Arévalo, Jorge Correa, Covadonga Salgado, Araceli E. Rossignoli, J. Pablo Lamas

**Affiliations:** 1 Centro de Investigacións Mariñas, Xunta de Galicia, Dirección Pedras de Corón, 36620 Vilanova de Arousa, Spain; araceli.escudeiro.rossignoli@xunta.gal; 2 Instituto Tecnolóxico para o Control de Medio Mariño, 36611 Vilagarcía de Arousa, Spain; amoronho@intecmar.gal (Á.M.); farevalo@intecmar.gal (F.A.); jcorrea@intecmar.gal (J.C.); csalgado@intecmar.gal (C.S.); plamas@intecmar.gal (J.P.L.)

**Keywords:** amnesic shellfish poisoning, ASP, multidecadal, prevalence, incidence, variability, cycles, mollusks, toxin, HAB, harmful algal bloom

## Abstract

Prevalence, impact on shellfish resources and interspecific, spatial, and temporal variabilities of domoic acid (DA) in bivalves from Galicia (NW Spain) have been studied based on more than 25 years of monitoring data. The maximum prevalence (samples in which DA was detected) (100%) and incidence (samples with DA levels above the regulatory limit) (97.4%) were recorded in *Pecten maximus*, and the minimum ones in *Mytilus galloprovincialis* (12.6 and 1.1%, respectively). The maximum DA concentrations were 663.9 mg kg^−1^ in *P. maximus* and 316 mg kg^−1^ in *Venerupis corrugata*. After excluding scallop *P. maximus* data, DA was found (prevalence) in 13.3% of bivalve samples, with 1.3% being over the regulatory limit. In general, the prevalence of this toxin decreased towards the North but not the magnitude of its episodes. The seasonal distribution was characterized by two maxima, in spring and autumn, with the later decreasing in intensity towards the north. DA levels decreased slightly over the studied period, although this decreasing trend was not linear. A cyclic pattern was observed in the interannual variability, with cycles of 4 and 11 years. Intoxication and detoxification rates were slower than those expected from laboratory experiments, suggesting the supply of DA during these phases plays an important role.

## 1. Introduction

In 1987, in Prince Edward Island, Canada, the intoxication of 104 people by consumption of mussels was reported [1,2]. The most striking symptom was the loss of short-term memory which led to naming the illness as amnesic shellfish poisoning (ASP). Later on, domoic acid (DA), a tricarboxylic amino acid, previously isolated from the red algae *Chondria armata* [3], and already known for its antihelmintic [3] and insecticidal properties [4], was identified as the main responsible agent [5]. Several isomers of DA have been described [6] but most of them seem to be less toxic than DA [7]. Bates, et al. [8] demonstrated that this toxin was produced in the area by the diatom *Nitzschia pungens* (now *Pseudo-nitzschia multiseries*). Since then, a number of diatom species have been shown to produce this toxin which has been found in many marine organisms all over the world [9,10,11,12,13].

Due to the evident risk that this toxin poses to human health, its maximum allowable level in food has been regulated in many countries. Additionally, chronic exposure to this toxin seems to have some effects in vertebrates [14,15], making its close monitoring even more important. Currently, in Europe and most countries, the maximum allowed concentration in shellfish is 20 mg kg^−1^ of meat [16,17,18,19].

Not all bivalve species have the same capability to accumulate DA because the two components of this process, absorption and depuration, are species-specific. The lower DA accumulation of the oyster *Crassostrea virginica*, in relation to the mussel, for example, is due to the rejection of *Pseudo-nitzschia* cells before ingestion [20]. The differences observed in other species can be due to the depuration rate. Most studied bivalve species depurate DA very fast [21,22,23,24,25] and consequently accumulate very little DA, while other species, such as the king scallop *Pecten maximus* [26,27,28] or the razor clam *Siliqua patula* [29,30,31] depurate slowly and can accumulate higher concentrations.

When DA concentrations in bivalves are over the regulatory limit, the harvesting (or marketing) of that species has to be interrupted, leading to direct and indirect economic problems (loss of income or distortion of culture cycle, for example) for fisheries and aquaculture. Several studies have suggested that the impact of toxic *Pseudo-nitzschia* blooms could be increasing due to climate change [32,33,34,35]

Galicia has the highest production of bivalves (particularly mussels) in Europe, with more than 238,000 metric tons per year since 2003 [36]. Extensive semicultures and natural populations of other bivalve species are also exploited (more than 500 metric tons per year since 2003), increasing additionally the biomass collected and the economic and social importance of the activity.

The actual impact of DA has not been evaluated in many commercially important bivalve species but it seems very important in some species with low DA depuration kinetics such as *P. maximus* [27]. In fact, in Europe it was necessary to enact the Decision 2002/226/CE to be able to commercialize at least the adductor muscle and gonad tissues of *P. maximus* [37] (because, in that species, removing the nonedible tissues, and especially the digestive gland, substantially reduces the DA concentration in the remaining tissues).

In order to minimize the risk of human intoxications and optimize monitoring systems it is necessary to know the main sources of variation of the toxicity in monitored bivalves, including the interspecific, spatial, and temporal variations. Temporal variation, additionally, has several components: (a) short-term variation, which is mostly a product of the balance between uptake and release of the toxins from bivalves (intoxication, detoxification); (b) seasonal variation; and (c) trend. There is little information about short-term variation in bivalves, and most of it deals with the intoxication/detoxification processes during single *Pseudo-nitzschia* blooms or with detoxification in laboratory conditions. The seasonal variation (of the toxic *Pseudo-nitzschia* populations or DA in bivalves) has been studied from several geographic locations but, usually, covering only one or only a few years [38,39,40,41,42,43,44,45]. Long-term variation (decadal or multidecadal) has also been studied but only on a few occasions [46,47,48].

In Galicia (Spain), DA in commercially important bivalves has been monitored by Intecmar using HPLC-UV since 1995. A large dataset has been gathered comprising more than 70,000 analysis which allows to describe adequately how the bivalves have been affected by DA as well as the possible trends and the spatial and seasonal variation.

**Figure 1:**
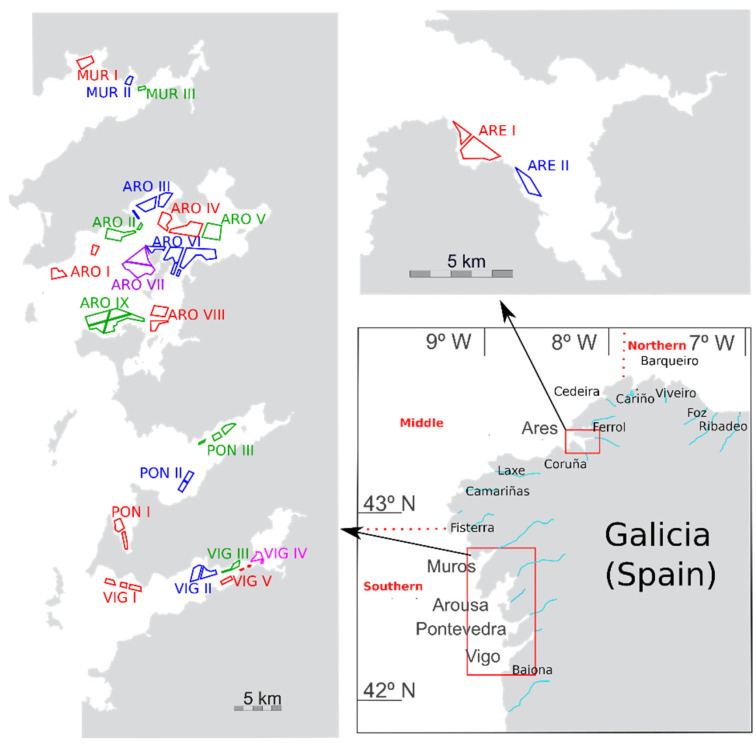
Area of study. Estuaries from which samples were taken (lower right panel), and mussel production areas (two other panels).

In this work, prevalence (proportion of the analyzed samples in which DA was detected), incidence (proportion of the samples with DA concentrations above 20 µg kg^−1^), and the main sources of variability of the DA concentration in bivalves of the area (Figure 1) have been characterized. This includes the apparent intoxication and detoxification rates of each species, the comparison of the levels in mussels (used in this case as the sentinel species) with other bivalves, and the analysis of the trends or cycles which might have taken place over a 25-year period.

## 2. Results

### 2.1. General

DA was detected in 11,157 samples out of 78,651 analyzed from 1995 until 2020. The proportion of this number corresponding to each bivalve species studied is shown in Table S1. Its general prevalence in the area was 14.3%. When the king scallop *P. maximus*—which always contained DA—was excluded from the analysis, the estimated prevalence was reduced to 13.3%.

Two point four percent of all samples tested were over the regulatory limit for DA. This reduced to 1.3% when the king scallop was excluded. The maximum level of DA in the area, 663.9 mg kg^−1^, was recorded in the king scallop *P. maximus.* Apart from this species, the maximum level detected was 316 mg kg^−1^ in *Venerupis corrugata*.

During DA outbreaks, its mean concentration was 14.9 mg kg^−1^ of mollusk meat, but only half of the observations (median) were above 3.89 mg kg^−1^. The top 5% concentrations were above 73.9 mg kg^−1^ (5% quantile). When the king scallop was excluded from the analysis, the mean was substantially reduced to 8.54 mg kg^−1^ and the median and 5% quantile, were also reduced to 3.4 and 32.3, respectively.

Studying those samples in which the relevant species (see Section 4.1) were analyzed during DA episodes, its concentrations showed a distribution approximately log-normal with a slight positive skew (Figure S1).

### 2.2. Interspecific Variation

**Figure 2:**
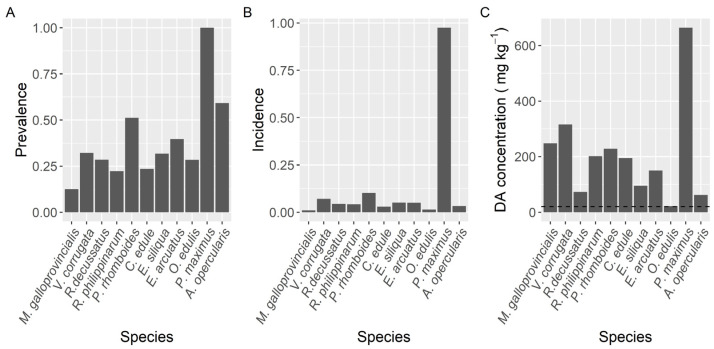
Proportion of the samples in which DA was detected (Prevalence) (**A**), in which the concentration was above the regulatory limit (Incidence) (**B**), and maximum DA concentration attained by the main bivalve species. The dashed line in panel (**C**) is the regulatory limit.

Prevalence of DA varied with the species studied (Figure 2). The highest prevalence (100%) was found in the king scallop *P. maximus* followed by *Aequipecten opercularis* (59%). Clams (*V. corrugata, Ruditapes philippinarum*, and *Polititapes rhomboides*), razor clams (*Ensis siliqua* and *E. arctuatus*), and cockle (*Cerastoderma edule*) showed intermediate levels of prevalence, ranging from 22 to 51%, and the minimum was observed in the mussel *M. galloprovincialis* (12.6%).

The proportion of samples whose concentration was higher than the regulatory limit (incidence) also differed substantially between species (Figure 2). It ranged from 1.1% in mussels to 97.4% in the king scallop (*P. maximus*)*,* with relatively high levels for two clam species *V. corrugata* and *P. rhomboides*, 7.0 and 10.2%, respectively.

The maximum DA levels in the area, 663.9 mg kg^−1^, were recorded in the king scallop *P. maximus*, followed by the clam *V. corrugata* (316.0 mg·kg^−1^) and the raft mussel *M. galloprovincialis* (248.5 mg kg^−1^) (Figure 2). The lowest maximum DA concentration per species (21.8 mg·kg^−1^) corresponded to the European oyster *O. edulis.* The average concentration during the DA episodes also varied with the species. The highest mean concentration corresponded to *P. maximus*. It was approximately fourfold higher than the levels observed in the clam *V. corrugata*, which had the second-highest average concentration. In the case of *P. maximus* both maximum DA levels and highest mean concentration per DA episode can be underestimated because in Galicia the harvesting of this species is only allowed below the requisites set up in the Commission Decision 2002/226/EC and accordingly samples of this species are only analyzed when: (1) there is not an active DA episode in the production area, (2) shellfish catchers request their exploitation, usually for Christmas and Easter and (3) DA concentrations in adductor muscle and gonad are below 4.6 mg kg^−1^.

**Figure 3:**
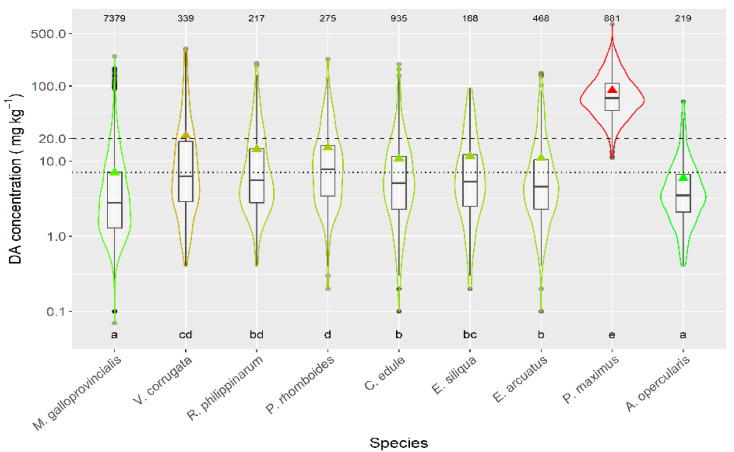
DA concentration during the toxic episodes in the main species of the area. Triangles = means, horizontal lines of the box = 25, 50, and 75% quantiles, extremes of the vertical lines from the box = range excluding outliers, dots = outliers. The outer shape (violin) represents the distribution of the data. The dashed line represents the regulatory threshold and the dotted one, the average level in raft mussels. The figures at the top of the plot are the number of observations. The averages of the species sharing the letters at the bottom of the plot were not significantly different (Tukey HSD test).

Taking into account the species whose mean DA concentration was computed from more than 50 DA quantified values, only the queen scallop (*A. opercularis*) had a mean concentration lower than that of the raft mussel, although this difference was not statistically significant. The means of all the other species of; clams, razor clams, and cockle were significantly higher than that of mussels (Figure 3).

Selecting only those data for which raft mussels and other bivalve species were collected from the same area, in the same week, the comparison between each individual species and raft mussels showed that the manila clam *R. philippinarum* and the cockle *C. edule*, had a significantly lower average DA concentration, while two clam species (*V. corrugata* and *P. rhomboides*), and especially *P. maximus*, accumulated more DA than mussels (Figure S2).

**Figure 4:**
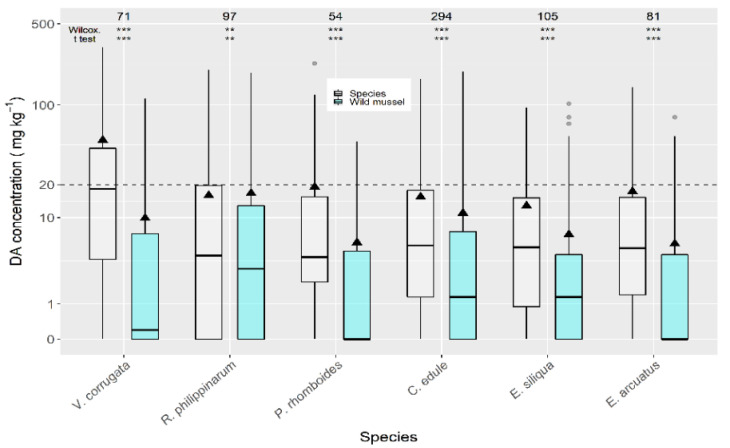
Comparison of DA concentration in wild mussel and other bivalve species, using data (maximum) collected from the same week and the same area. The asterisks are the significance levels estimated by paired Wilcoxon and *t* tests. All other symbols as in Figure 3.

When compared to wild mussels (also sampled from the same estuary in the same week) the average concentrations of all the analyzed bivalve species were higher (Figure 4). The highest difference was recorded for *P. maximus* (not shown) which had DA levels substantially higher than wild mussels. The next largest differences were recorded for the clams *V. corrugata* and *P. rhomboides*. The manila clam and the cockle showed the lowest differences.

DA concentrations over the regulatory limit in wild mussels, *C. edule* (20%), and *E. siliqua* (15%) were below those for *V. corrugata* (35%) from the same week and production area.

The frequency distributions of the DA concentration recorded during the episodes, in the different species studied, are approximately log-normal, in some cases with a positive skew and, in some other, with a positive kurtosis (leptokurtic, Figure S3).

### 2.3. Spatial Variation

**Figure 5:**
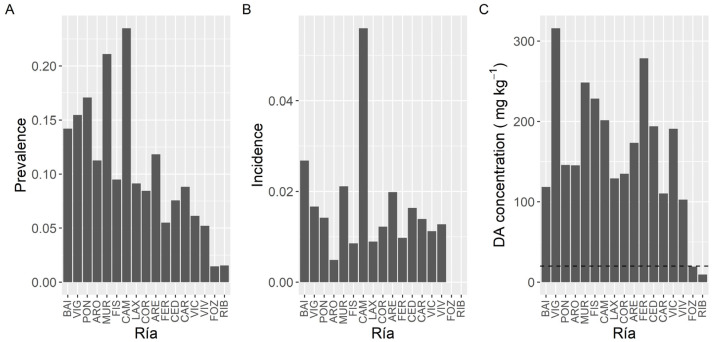
Proportion of the samples in which DA was detected (Prevalence) (**A**), in which the concentration was above the regulatory limit (Incidence) (**B**), and maximum DA concentration attained by the bivalves in each estuary. The dashed line in panel (**C**) is the regulatory limit.

Once *P. maximus* was excluded from the analysis (because it has a very high prevalence and it is collected mostly from only one estuary) DA prevalence showed an increasing trend from the southernmost estuaries towards Camariñas (CAM), and then a decreasing one up to Ribadeo (RIB), in the northeast extreme of the sampling area (Figure 5). The maximum prevalence levels, which were slightly over 20%, were observed in the estuaries of Camariñas and Muros-Noia (MUR), and the minimum ones, below 3%, in the estuaries of Ribadeo and Foz.

In contrast to prevalence, incidence did not show a clear geographical pattern (Figure 5). The highest incidence corresponded to the Camariñas estuary, followed by the Baiona estuary (BAI) and the estuaries of Vigo (VIG), Muros-Noia and Ares-Betanzos (ARE). The minimum levels were recorded in the easternmost sampling location, the Ribadeo estuary.

Likewise, maximum DA concentrations did not show a clear geographical trend (Figure 5). The highest maximum levels were recorded in bivalves from the Vigo estuary, followed by the Ferrol (FER) estuary, Fisterra (FIS), and the two sampling locations nearby. Lowest maximum levels corresponded to the Ribadeo estuary, followed by the Foz estuary, both in the Cantabrian Sea.

**Figure 6:**
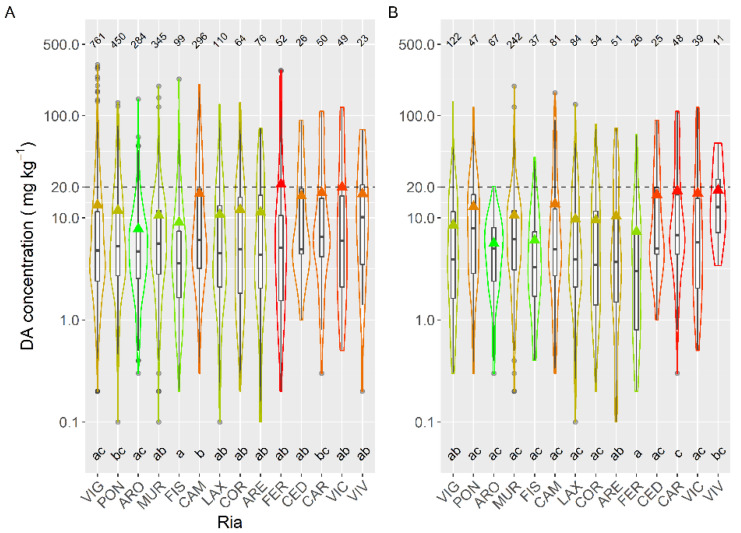
DA concentration in the main bivalve species with the exception of king scallops and mussels (**A**), and in the cockle (**B**), during the toxic episodes in the Galician estuaries. All symbols as in Figure 3.

To study the concentration of DA during the episodes, mussels and king scallops were initially excluded from the analysis. Mussels were excluded to eliminate both, the bias due to the presence or not of mussel rafts (mussel culture in rafts only exist in five estuaries, four of them in the southernmost area), and the fact that wild mussels are not usually sampled during the episodes, because in Galicia this species cannot be usually commercialized. Thereafter DA average concentration varied with the location, and the differences between estuaries were, in general, relatively small (from 7.8 to 20.0 mg kg^−1^) (Figure 6). Apart from the estuaries of Ribadeo and Foz (in which a small number of samples of species other than mussel (wild) have been obtained), the lowest average concentration was detected in the Arousa estuary (ARO), which also had the minimum incidence but not the minimum prevalence or the lowest maximum DA level. Otherwise, the highest average value was found in the Ferrol estuary, which also had the maximum DA concentration recorded but neither the maximum prevalence nor the maximum incidence.

When mussels were included in the spatial variation analyses, the observed pattern was very similar to that shown in Figure 6.

If the data corresponding to the cockle—which was the most sampled species after the mussel—are used, the relative importance of some estuaries varies considerably. Cockles from the Ferrol estuary had a substantially lower average concentration than the whole bivalve population, and the opposite happens in Pontevedra.

**Figure 7:**
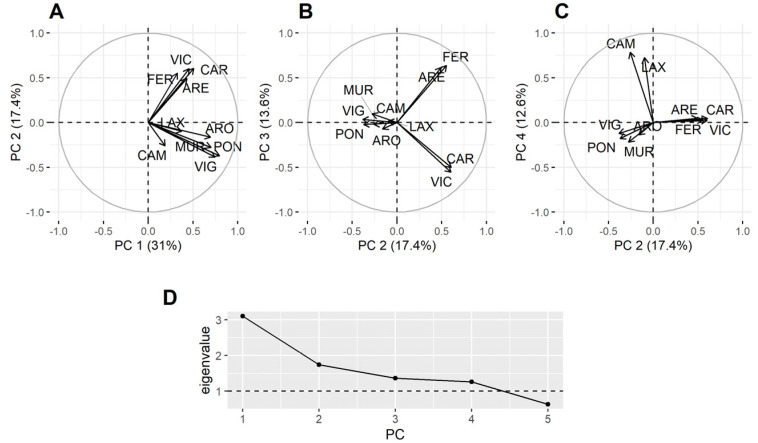
Loadings of the four principal components and their corresponding eigenvalues. All data but those corresponding to the king scallop were included (**A**–**D**).

Principal component analysis (PCA), using all data but excluding *P. maximus*, showed that not all estuaries behaved in the same way in relation to the DA concentration in bivalves (Figure 7). The first component reflected the general covariation, but the second separates the southernmost estuaries from the northernmost ones (some estuaries have not been included in the analysis because they have a high number of missing data). The third component separates the northern estuaries into those in the Atlantic and the Cantabrian coasts. The fourth one separates the estuaries of Corme-Laxe (LAX) and Camariñas, which are located north of Cape Finisterre, from all others.

In order to check if the results obtained were due to the predominance of raft-cultured mussel samples in southern estuaries, a new PCA, excluding *P. maximus* and raft mussels, was carried out. As in the previous analysis some estuaries had to be excluded because they have a high number of missing data or because the available data were not well synchronized with the other estuaries (in this case, all southern estuaries but Vigo (VIG) were excluded). The second component separated the northernmost estuaries into two groups, one in the Atlantic and another in the Cantabrian coast. The third component separates the southern estuaries (positive loadings from the northern ones) and the fourth component separates the southern estuaries into two groups: located to north and south of Cape Finisterre (Figure S4).

**Figure 8:**
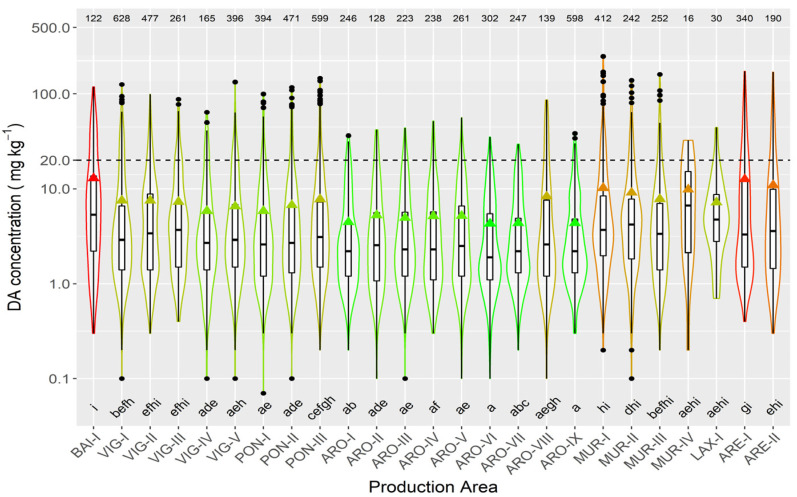
DA concentration in the main bivalve species with the exception of raft mussels, during the toxic episodes in the Galician estuaries. All symbols as in Figure 3.

In raft-cultured mussels, the highest mean concentration levels were recorded in the two production areas of the Ares-Betanzos estuary and Baiona. The lowest values were found in most of the production areas of the Arousa estuary (Figure 8).

### 2.4. Seasonality

**Figure 9:**
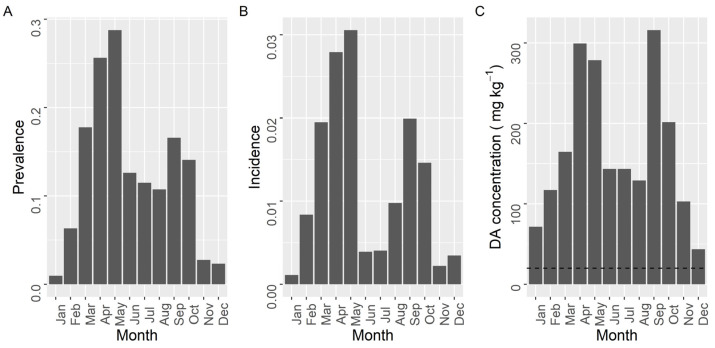
Proportion of the samples in which DA was detected (Prevalence) (**A**), in which the concentration was above the regulatory limit (Incidence) (**B**), and maximum DA concentration attained by the bivalves in each month of the year. The dashed line in panel (**C**) is the regulatory limit.

The prevalence, incidence, and maximum concentration of DA, presented two maxima during the year (Figure 9). The first took place in spring (March for the two first variables and April for the third) and the second at the end of summer (September). The minimal values of prevalence and incidence were recorded from November to January. A secondary minimum, that was especially intense for incidence, was observed during the summer months (June–July). The maximum concentration showed a similar seasonal pattern but winter and summer minima, especially the former, were much less pronounced than minima for prevalence and incidence.

**Figure 10:**
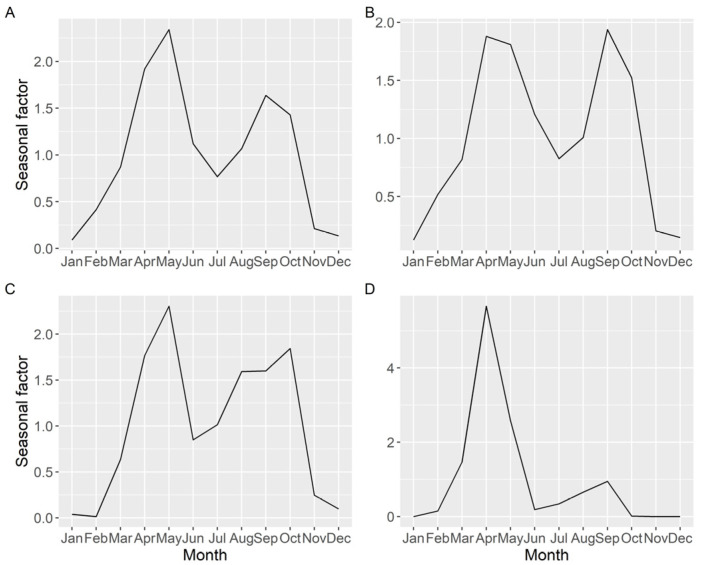
Seasonal pattern (ratio of actual value:deseasonalized value), of the average DA concentration, obtained by time series analysis for all the Galician (**A**), southern (**B**), middle (**C**), and northern estuaries (**D**).

Seasonality of the average DA concentration (including samples with zero concentration), estimated by time series analysis, showed two peaks, one in spring and another in autumn, but the relative importance of each peak varied with location (Figure 10). The more to the north the estuary is located, the less intense was the autumn peak.

**Figure 11:**
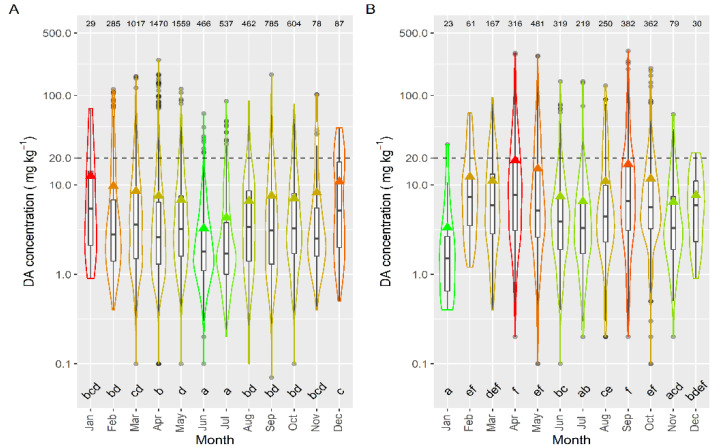
DA concentration over the year in raft mussels (**A**) and the other main bivalve species (**B**), during the toxic episodes in the Galician estuaries. All symbols as in Figure 3.

The intensity of the toxic episodes of DA in raft-cultured mussels showed a concentration maximum in winter months—December and January—descending progressively until June, and then ascending until December (Figure 11). Interestingly, even when winter DA levels in mussels were at their highest, the number of samples in which DA was detected was smaller than those in most other months (except November). Most mussel samples were obtained from rafts, which are located mostly in the southernmost estuaries, and consequently, the observed pattern is biased towards the seasonal pattern in these estuaries.

The seasonal pattern, when mussels were excluded from the analysis, was more consistent with the observations of prevalence, incidence, and maximum concentration. It showed April and September maxima and January and June–July minima (Figure 11). These observations are more independent of the south–north differences in mussel samples.

### 2.5. Time Course of the DA Concentration in Different Locations

**Figure 12:**
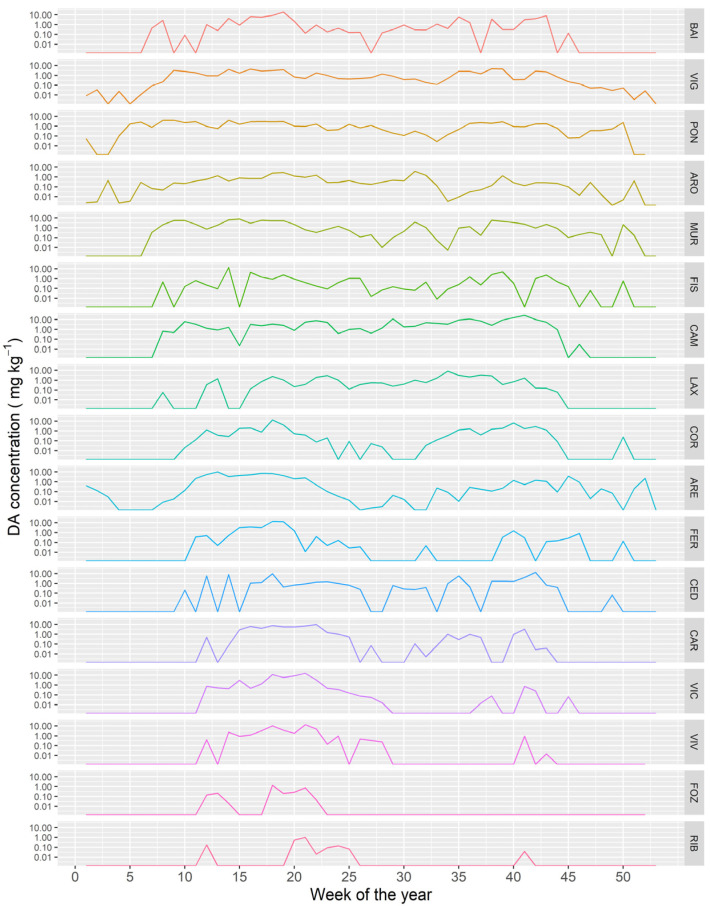
Average DA concentration along the year in the sampling locations along the Galician coast, from the southernmost ones (upper panels) to those in the extreme northeast (lower panel).

The time course of the DA concentration varied between the different Galician estuaries. As a general trend, the more to the northeast the estuary is located (from the southern part of the Atlantic margin to the eastern part in the Cantabrian Sea) the shorter the period in which the bivalves were affected by DA (Figure 12).

### 2.6. Interannual Differences

**Figure 13:**
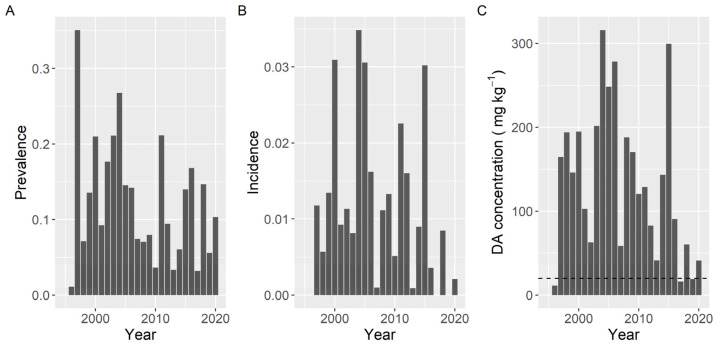
Proportion of the samples in which DA was detected (Prevalence) (**A**), in which the concentration was above the regulatory threshold (Incidence) (**B**), and maximum DA concentration attained by the bivalves in each year. The dashed line in panel (**C**) is the regulatory limit.

Variation of prevalence, incidence, and maximum concentration along the sampling period did not show the same pattern. The observed differences in prevalence were smaller than those for the other two variables. The three variables presented a peak in 2004. Prevalence and incidence also showed a common peak in 2011, and incidence and maximum level also had high values in 2005 and 2015 (Figure 13).

**Figure 14:**
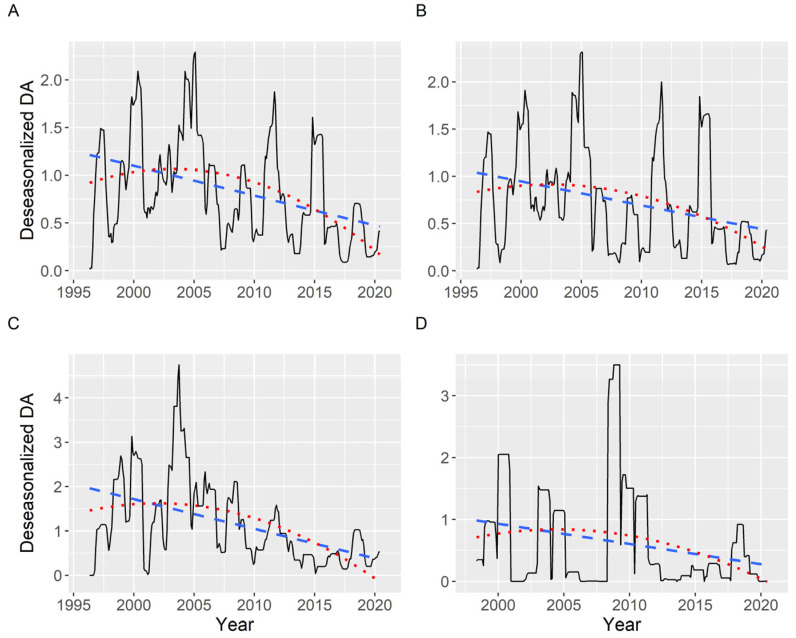
Deseasonalized average DA concentration corresponding to all (**A**), southern (**B**), middle (**C**), and northern estuaries (**D**). The dashed and the dotted lines are the linear and quadratic trend, respectively, fitted by regression.

Once deseasonalized by time series analysis, a slightly decreasing trend has been detected when the average DA concentration (including zeros) was examined. The trend is not strictly linear, as a quadratic curve fits it better than a straight line (statistically significant effect). The R^2^ was, notwithstanding, low, ranging from 0.08 to 0.30 and being, in general, 0.22. This trend was similar in all the areas studied (Figure 14).

The partial autocorrelation of the time series (including zeros), when all data were analyzed, showed two main periodicities: 4 and 11 years. The 4-year periodicity was also observed when the southern, medium, and northern estuaries were analyzed separately, but the 11-year periodicity was restricted to the southern estuaries (Figure S5).

**Figure 15:**
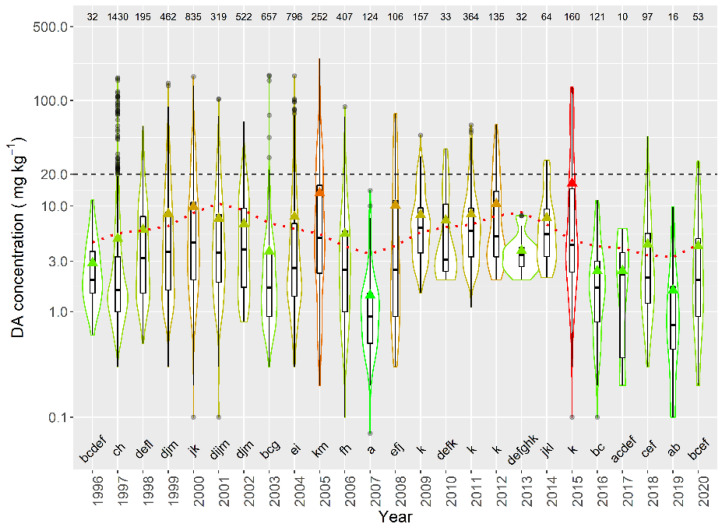
DA concentration in the episodes along the sampling period in raft mussels. The dotted line is the result of fitting the combination of a linear decrease and two sinusoids with periods of 4 and 11 years. All other symbols as in Figure 3.

When the average concentration per episode in raft-cultured mussels was studied, large differences in DA concentration were detected between years, over the sampled period. Average concentrations below 3 mg kg^−1^ were detected in 1996, 2007, 2016, 2017, and 2019, while the maximum levels were recorded in 2005 and 2015. In general, the periodicities and time trend observed for the average concentration (decreasing trend and cycles of 4 and 11 years) seem to also apply for the magnitude of the episodes. A combination of two sinusoidal oscillations with periods of 11 and 4 years and a linear trend was fitted to the data by regression, its cosine component being statistically significant (*p* = 0.03) (Figure 15).

### 2.7. Apparent Intoxication and Detoxification

The apparent intoxication and detoxification rates were computed from the change in DA concentration in bivalves recorded between two consecutive weeks. The true rates cannot be computed because, while mollusks are increasing its toxins content, depuration is simultaneously occurring, and consequently the observed increase is in fact the absorbed toxin minus the depurated one. The opposite happens during the depuration period, in which the depurated toxin would be underestimated because some toxic cells could still be ingested by the bivalves. With this approach, therefore, only apparent rates, and not true ones, can be computed.

**Figure 16:**
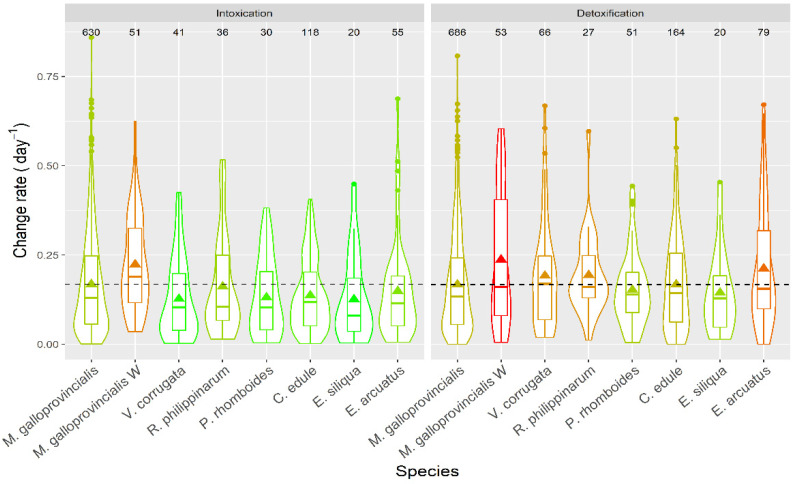
DA apparent intoxication and detoxification rates in the main bivalve species of the Galician estuaries. The dashed line represents the levels corresponding to the raft mussels. The number in the upper part of the figure are the number of observations. All other symbols as in Figure 3.

Apparent intoxication rates were, in general, low (Figure 16), with averages below 0.25 day^−1^, which means a doubling of DA concentration in the bivalves would take, on average, ~3 days. With the highest intoxication rates recorded, notwithstanding, this time would be less than one day. The apparent depuration in all species was not very fast, with average levels below 0.25 day^−1^, and rarely reaching 0.5 day^−1^.

## 3. Discussion

### 3.1. Interspecific Variation

The presence of DA in bivalves from Galicia is relatively frequent. In more than 13% of the samples obtained by the monitoring system from 1995 to 2020, DA was detected. Prevalence was not the same for all species. DA was found in all samples of *P. maximus* analyzed but in 12.6% of those of *M. galloprovincialis*. All other species showed intermediate values between 22 and 59%, but these prevalences can be slightly overestimated in relation to mussels because their sampling was less frequent and focused on toxin outbreaks.

Prevalence data of DA in bivalves are rarely reported. A higher prevalence than that in Galicia has been observed in Scotland between 2003 and 2004 where DA was detected in 56% of the samples (excluding king scallop) [49], and in Catalonia (NE Spain) 23.8% [50]. Lower prevalence, below 15%, has been observed in Greece during 2002 and 2003 [51] and in Wales and England, from 1999 to 2009, 2.4% [52]. Prevalence data, in any case, may not be comparable among different reports as they would depend on the limit of detection of the methods used. Prevalence also depends on the characteristics of each bivalve species. DA is expected to have a higher prevalence in species with a low depuration rate, as is the case of *P. maximus*, because the toxin would persist in the bivalve well after the toxic bloom has ended [26,27] while, in species with fast depuration, the detection of the toxin would be more restricted to the time when the blooms take place.

The impact of DA on the harvesting of bivalves was much smaller than its prevalence. On average (excluding the king scallop *P. maximus*) only 1.3% of the total samples had concentrations above the regulatory limit. Thus, the impact, in general, is very limited when compared to that of lipophilic toxins from the same area, which was ~10.3%, one order of magnitude higher, during the period 2014–2017 [53,54], but higher than in other places of the Iberian Peninsula, like Portugal or Catalonia [50]. DA concentration in king scallops was above the regulatory limit in 97.4% of the samples, which was clearly due to the low DA depuration by this species [26,27,28,55]. A high incidence of DA in this species has been observed in several locations [26,27,28,56,57,58,59,60,61,62].

The maximum DA concentration in the whole body, 663.9 mg kg^−1^, was recorded in *P. maximus*. This concentration was lower than that recorded in Scotland, 1,569 mg kg^−1^ [60], in the Atlantic coast of Andalucía (South Spain), 980 mg kg^−1^, and France, 861 mg kg^−1^, [63], but higher than that reported from Ireland, 154.3 mg kg^−1^ [57].

The second and third species with the highest DA concentration were the clam *V. corrugata*, 316 mg kg^−1^, and the mussel *M. galloprovincialis*, 248 mg kg^−1^. These maximum levels were higher than in Portugal (cited as *V. pullastra* and *M. edulis*). The value of *M. galloprovincialis*, as far as we know, is the highest recorded for that species, with concentrations substantially lower in Greece [51], Turkey [45], Croatia [64], and the Mediterranean coast of Spain [65]. A similar species was responsible for very high toxin levels in Prince Edward Island (Canada), 790 mg kg^−1^, and the UK, 450 mg kg^−1^ [63]. The European oyster *O. edulis* had the lowest maximum DA concentration among the species studied, 21.8 mg kg^−1^, a characteristic which seems to be constant in most countries in which it was studied [50,63,64]. Little information is available for *V. corrugata*. The maximum level reported from Portugal was 17 mg kg^−1^ [66].

The magnitude of the episodes was also dependent on the species. When the magnitude was measured by average DA concentration, then its highest value was recorded in *P. maximus*, followed by. *V. corrugata*, *P. rhomboides*, *R. philippinarum*, *E. siliqua*, *C. edule*, *E. arctuatus*, *M. galloprovincialis*, and *A. opercularis*, in this order. When magnitude was measured by the maximum concentration attained, *P. maximus* still had the highest value, but the order of the other species was not the same: *V. corrugata*, *M. galloprovincialis*, *P. rhomboides*, *R. philippinarum*, *C. edule*, *E. arctuatus*, *E. siliqua*, and *A. opercularis*. The most important difference is that the magnitude of the *M. galloprovincialis* episodes is much higher, relative to the other species, when maximum levels were used. The most likely explanation would be that this species is the less associated with the benthic area (mostly in mussel rafts) and, consequently, toxic algae are available in a different way than for the benthic species. Mussel rafts receive toxic cells directly from the planktonic populations, so they can ingest a large amount of toxins, and attain high maximum levels, but, when the populations decay, the toxin supply disappears. On the contrary, the toxic cells are probably less available for the benthic species during the early phases of blooms (e.g., by boundary layers, or tidal cycles), but the toxic populations can accumulate at the bottom and constitute a more stable toxin source.

### 3.2. Relation between DA Concentration in Mussels and Other Bivalves

Mussels are frequently used as indicator species for different contaminants [67,68]. In the Galician monitoring system, they are used as sentinel species. Two kinds of mussels —raft-cultured and wild—have to be used in this study because the two types are not sampled in all areas. After comparing DA concentrations in raft mussels with those obtained in the same area and the same week for other bivalve species, several important species, such as *V. corrugata* and *P. rhomboides*, showed DA levels significantly higher, and *R. philippinarum* and *C. edule* significantly lower. Wild mussels were even less useful to predict DA levels in other bivalves because all the studied species showed significantly higher concentrations. Recently, Rourke et al. [69] came to the same conclusion with another mussel species (*M. edulis*). This suggests that although mussels may not be a good indicator species for reducing sampling of other bivalves, the presence of DA in mussels indicates a requirement to test other bivalves from the same production area. To maintain the safety for human consumption during the period between mussel and bivalve analysis, administrative decisions, as precautionary closures of the production area, should be implemented. 

### 3.3. Spatial Variation

The northern estuaries had less prevalence than the southern estuary but the incidence was approximately the same, except for the easternmost estuaries (Foz and Ribadeo) in which both variables had very low values. Average DA concentration during the episodes was higher in the northern estuaries (again except Foz and Ribadeo).

It seems clear from the PCA that the Galician estuaries can be separated into three groups by their DA concentrations in bivalves: estuaries to the south of Cape Finisterre, from Finisterre to Cape Ortegal, and from Cape Ortegal to the east. This geographic classification coincides with the orientation of the coast.

The low impact of DA in Foz and Ribadeo can be due, not only to their location (more northeastern) but also to their morphology. In two estuaries (purely estuarine), freshwater is much more important than in the other estuaries.

Regarding the production areas where mussel is cultured in rafts, most areas in the Arousa estuary, followed by those in Pontevedra estuary, had episodes with the lowest average DA concentration. The Ares-Betanzos and Baiona estuaries had the most intense episodes.

### 3.4. Seasonality and Timing of the Episodes

DA prevalence, incidence, maximum, and average concentration followed a clear seasonal pattern with two maxima, the first in spring and the second in autumn, and two minima corresponding to winter (main) and summer. In autumn, DA contaminations were less frequent and had reduced impacts on bivalve fisheries and aquaculture however, toxicity levels could reach the same magnitude as those observed in spring. The seasonal pattern was not the same for all estuaries. The further North the estuaries are, the lesser relative importance of the autumn maximum.

In the Atlantic Ocean, the presence of a spring peak of DA is frequent. The secondary maximum, notwithstanding, is usually much smaller than the first one, as in Aveiro (Portugal), for example [70]. In Galicia the main episode occurs in the summer, however, in some places, it takes place earlier [39,46], while in Scotland the main episode occurs in autumn [55].

In some areas of the Mediterranean Sea, the main DA peak seems to take place in summer and a secondary in autumn [41,45], but not in others where the maximum incidences took place between February and August [50].

In the Southern California Bight the seasonal pattern is similar to that detected in the northern estuaries, with a maximum in spring and a smaller secondary maximum (which takes place in summer instead of autumn) [47]. In that area, as in Galicia, north–south differences exist, but in California the relative importance of the secondary maximum decreases to the south, nearly disappearing [47]. In Vostok Bay [42], in the Japan Sea, and in Vietnam [38], the seasonality differs, with the presence of DA in bivalves during autumn–winter.

The average DA concentration during the detected episodes followed the same pattern, with two maxima in spring and autumn, when raft mussels were excluded from the analysis. In raft-cultured mussels there was a maximum in winter and a minimum in summer. The number of episodes recorded is, anyhow, low. This special pattern is probably due to short-lived planktonic blooms that are intense, and perhaps patchy, hence they do not reach the intertidal zone.

The timing of the DA episodes also showed a trend in the south–north direction. The further north production areas are, the shorter the episodes.

### 3.5. Interannual Variability

The interannual variability of DA in bivalves was high. The recorded variability of the incidence was higher than that of the prevalence and the maximum and average concentration. All those variables have decreased since 1995 but it seems that not in a linear way, having a maximum around 2004–2005. A decreasing trend was also observed in DA presence in bivalves from England and Wales, from 1999 to 2009, even when the trend in the *Pseudo-nitzschia* populations was the reverse [52].

The periods with the highest DA levels in Galicia (2004–2005 and 2011–2014), coincided with some in other geographic areas. High DA levels were recorded in 2004 in the nearby Portugal [70] but not in the Atlantic coast of France [46], in the Mediterranean coast of Spain [50], or in California [47]. High levels in 2011–2014, have been recorded in California [47].

Some cyclic behavior in average concentration was also detected. A 4-year cycle seems to be common to all studied estuaries, and an 11-year cycle seems to be present in the southernmost estuaries. The same cycles seem to be present in the average concentration during the episodes. An 11-year cycle was also found in solar activity and climate [71], and Vale [72] has suggested that solar activity can impact PSP episodes in the Portuguese and Galician coasts. A direct link of solar activity with the cycle observed in this study seems unlikely because it was only detected in the southern estuaries, and not in the middle and northern estuaries.

Four-year cycles have been described for several variables in the marine environment. A 3–5 year Chl *a* cycle in the northeast Atlantic [73], and 4-year cycle in the paleontological records of *Skeletonema costatum* from British Columbia [74] were found, but their causes are unclear. A cycle with the same periodicity, possibly linked to the North Atlantic Oscillation and the East Atlantic Pattern [75], was also found for the recruitment area of the sardine *Sardina pilchardus*.

### 3.6. Intoxication and Detoxification Velocity

Apparent intoxication rates varied with the species. Intoxication rates were higher in mussels than in all other species. This may be due to several causes: (a) bivalves may take toxic cells at different rates, depending on their clearance rates, (b) pre- or postingestive selection against the toxic cells, (c) reduction of the absorption efficiency derived from an increase of the concentration of available particles; and (d) different availability of toxic algae in the areas where the different bivalves grow. Cause (a) could explain the rates of some species, such as *C. edule* [76] or *R. philippinarum* [77] which have clearance rates lower than *M. galloprovincialis* [78] or the other clam species, *P. rhomboides* (as they share the same morphology and they are expected to have similar pumping rates per gill area [79]). It cannot explain, nevertheless, the rates of other species which have higher clearance rates, such as *A. opercularis* [80,81], or approximately equivalent, such as *V. corrugata* [82] and the *Ensis* species (if their rates are similar to those of *E. directus* [83], the only species in the genus for which they have been reported). Cause (b), the pre- or postingestive selection against the toxic cells (shown to be the main cause of difference in DA uptake between oysters and mussels [20,84]) cannot be demonstrated in this case because no data about on the rejection of *Pseudo-nitzschia* cells by the studied species are currently available. Cause (c), the reduction of the absorption efficiency, derived from an increase in the concentration of available particles, is possible because mussels grow in rafts or in rocky shores, where the amount of suspended inorganic particles is expected to be substantially less than in sandy or muddy environments. The quality of the food that the mussels receive is expected to be higher than other mollusks, and, consequently, the gut passage time should be longer [85,86,87] and the absorption efficiency higher. Cause (d), the different availability of toxic algae in the areas where the different bivalves grow, seems unlikely because the recorded differences between wild mussels and all other bivalves were larger than with the raft mussels while its environment is more similar.

The observed detoxification rates were lower than expected from laboratory experiments. *M. galloprovincialis* has been shown to depurate DA at a rate of 0.4–0.58 day^−1^ [22], *R. philippinarum* at 0.44 day^−1^ [88], and *P. rhomboides* and *V. corrugata* at 0.49 and 0.75 day^−1^, respectively (unpublished data). These differences may be due to some uptake of DA during the depuration phase, which reduces the apparent depuration speed. Taking into account that DA depuration of wild mussels, the only studied organisms that inhabit the rocky shore, is faster than that of the other bivalves, it seems likely that the bivalves from muddy or sandy environments may ingest DA from sedimented cells or even from sediments to which it can be adsorbed [89].

### 3.7. Intersample Variability

The general data distribution was approximately log-normal. There is some skewness which may be due to the LOQ data, which fulfill both retention time and wavelength spectra requisites, being included in the analysis. The overall coefficient of variation was 65%, varying from 0.31 in *A. opercularis* to 78% in wild mussels.

## 4. Materials and Methods

### 4.1. Sampling

Samples are collected with a minimum weekly frequency, for all the production areas of Galicia in which harvesting of bivalve mollusks is allowed (depending on the exploitation plans). The mussel *Mytilus galloprovincialis* (culture in rafts or wild) is used as a sentinel species. When a toxic episode is detected in mussels, other harvested species (cockles, clams, oysters, razor-clams, and queen scallops) are sampled and analyzed. Harvesting of wild mussels for human consumption is not usually permitted in Galicia (except in restricted production areas). In some locations, it is used as a sentinel for infaunal species, but since the onset of a toxic episode is detected, the infaunal species are analyzed and wild mussels are not newly analyzed until that DA episode is finalized. The level of ASP toxins in scallops, *P. maximus*, is usually above the legal limit, in all production areas of Galicia, hence their harvesting is only allowed for those production areas that fulfill the conditions established in the annex of the Decision 2002/226/CE. Sampling and analysis of scallops is then limited to short periods, when harvesters are interested in their exploitation, usually during Christmas or Easter.

### 4.2. Reagents and Reference Solutions

Due to the long sampling period, different reagents have been supplied by different vendors but purity grade was maintained. Reference solutions of different batches of two suppliers were also used. Reagents: Hydrochloric acid (37%) ACS reagent grade, Acetonitrile HPLC grade, Glacial Acetic acid ACS reagent grade, Milli-Q water (Millipore Ibérica, Madrid, Spain).

Domoic acid reference solutions: Certified standards of DA were purchased from both National Research Council of Canada (B3H 3Z1, Institute for Marine Biosciences, Halifax, Nova Scotia, Canada) and CIFGA laboratory S.A. (Lugo, Spain).

### 4.3. Extraction

DA was extracted following the Lawrence et al., procedure [90] in order to share the same extracts for DA analysis and PSP bioassays. Bivalve mollusks were opened and the shells discarded. Soft tissues were pooled and homogenized with a blade blender. A 100-g aliquot of the homogenate was mixed with 100 mL of HCl 0.15 N, and the pH adjusted to 3 (range 2.5 to 3.5). The mixture was boiled for 5 min, and let to cool to room temperature, adjusting both, the volume lost by evaporation and the pH (between 2.5 and 3), if necessary. To clarify the solution, 20 mL of the extract was transferred to a test tube and centrifuged for 5 min at 4000× *g*. Finally, a 1 mL aliquot of the supernatant was filtered through a 0.22 µm syringe filter into a HPLC vial for LC-DAD analysis.

### 4.4. Analysis

Analyses were performed with a Waters Alliance (2690 separations module and column oven, and 996 diode array detector, set at 242 nm) (Waters Cromatografía SA, Cerdanyola del Vallès, Spain) in isocratic mode, with different C18 (L1) columns of 125 mm × 4.6 mm, and 5 µm of particle size, kept at 30 °C. The mobile phase used was 1% AcOH: CH_3_CN (90:10 v:v), and the flow rate was between 0.6 and 0.8 mL min^−1^, depending on the column. The samples were kept at 10 °C and injected (20 µL) in less than 4 h after the extraction.

Samples were quantified using domoic acid certified reference materials.

### 4.5. Data Processing

Data were processed in two ways: one to monitor the presence of DA in the bivalves, and the other to characterize the toxic episodes. In the first case, all samples were used, in the second, only those samples which contained detectable levels of DA were used.

#### 4.5.1. Monitoring DA in Bivalves

With this aim, prevalence (proportion of the analyzed samples in which DA was detected), incidence (proportion of the samples with DA concentrations above 20 µg kg^−1^), principal component (to explore the spatial variability), and time series analysis (to explore the temporal variability, including trend, seasonal variation, and autocorrelation) were used.

In all cases, except for their distribution among species, the scallop *P. maximus* was excluded from the analysis because of its permanent contamination, its infrequent sampling, and its locally restricted distribution.

Principal component analyses were carried out using the logarithmically transformed data (log(DA + 1)), to avoid losing the data with zero concentration), utilizing the R packages FactoMineR and factoextra. Two sets of data were used to carry out the analyses. The first one included all the obtained data excluding the king scallop. The second one, additionally excluded raft cultured mussels, to eliminate the possible bias introduced by the high number of such samples.

Time series analysis was carried on all the available data, with the exception of the scallop *P. maximus*. The R packages stat, forecast, and t series, were used for the analysis, and a multiplicative model was used for the decomposition.

#### 4.5.2. Characteristics of the Toxic Episodes

The characteristics of the episodes were described by statistics (mean, median, quartiles, range, and shape of the frequency distribution) using a combination of violin and boxplots. The R-package ggplot2 was used. The statistical differences between species, locations (estuaries for all species and production areas inside estuaries for raft-culture mussels), and time (month and year) were estimated by means of ANOVA and Tukey HSD a posteriori tests. *P. maximus* was excluded from the analysis other than the differences between species, and, when comparing estuaries, two additional datasets were used, one excluding additionally the mussels (to check the influence of mussel rafts), and another including only the cockle *C. edule*, which was sampled from most estuaries. The locations with less than 10 observations were excluded from the analysis.

#### 4.5.3. Comparison between Mussels and Other Species

The average DA concentrations of the episodes in raft-cultured and natural bed mussels were compared with those corresponding to the other species studied. The datasets for these comparisons were prepared by choosing, for each bivalve species, the samples which have a corresponding sample of mussel (raft or natural bed) in the same estuary and the same week. When several samples from the same combination of species/estuary/week existed, the maximum value was used. Once all the pairs of mussel-species data were obtained, the statistical differences were checked by means of paired Student *t*-tests applied to the logarithmically transformed data (log(x + 1)), and by nonparametric paired Wilcoxon tests, both from the stats package of R.

#### 4.5.4. Frequency Distribution

To estimate the frequency distribution of DA in bivalves, the residuals of the ANOVA of log(DA concentration) with species and estuary were used. Some species were not included in the analysis: the scallop *P. maximus* because of its permanent contamination with DA, the oyster *M. gigas*, because of its low number of observations, and the mussels collected from natural beds, because they usually were not sampled after its concentration reached the regulatory limit. The estimation of the distribution by this method is an approximation that can be biased because it includes the variation with time into the error. To check if the bias is important the residuals of the other ANOVA possible, log(DA concentration) with species and week, were used (thus including the variation with location into the error).

## Data Availability

This study (PSTs) and Intecmar (under request).

## Supplementary Materials

The following are available online at https://www.mdpi.com/article/10.3390/toxins13110756/s1, Figure S1: Frequency distribution of the DA concentrations; Figure S2: Comparison of DA concentration in raft mussel and other bivalve species; Figure S3: Frequency distribution of DA concentrations by species; Figure S4: Loadings of the four principal components and their corresponding eigenvalues after excluding king scallops and raft mussels from the analysis; Figure S5: Partial autocorrelation plots. Table S1: Percentage of the total number of samples analyzed corresponding to each bivalve species.
