# Supplementary material for: Twenty-Five Years of Domoic Acid Monitoring in Galicia (NW Spain): Spatial, Temporal, and Interspecific Variations
Source: Toxins (Basel). 2021 Oct 25;13(11):756. doi: 10.3390/toxins13110756 (PMC8624277; doi:10.3390/toxins13110756)
Supplement: Supplementary file 1 [file toxins-13-00756-s001.zip › toxins-1391926 - Supplementary.pdf]

toxins

MDPI

Supplementary Information

Twenty-Five Years of Domoic Acid Monitoring in Galicia (NW Spain): Spatial, Temporal, and Interspecific Variations

Juan Blanco [1,*], Ángeles Moroño [2], Fabiola Arévalo [2], Jorge Correa [2], Covadonga Salgado [2], Araceli E. Rossignoli [1] and J. Pablo Lamas [2]

[1] Centro de Investigacións Mariñas, Xunta de Galicia, Dirección Pedras de Corón , 36620 Vilanova de Arousa, Spain; araceli.escudeiro.rossignoli@xunta.gal

[2] Instituto Tecnolóxico para o Control de Medio Mariño, 36611 Vilagarcía de Arousa, Spain; amoronho@intecmar.gal (A.M.); farevalo@intecmar.gal (F.A.); jcorrea@intecmar.gal (J.C.); csalgado@intecmar.gal (C.S.); plamas@intecmar.gal (J.P.L.)

* Correspondence: juan.carlos.blanco.perez@xunta.gal

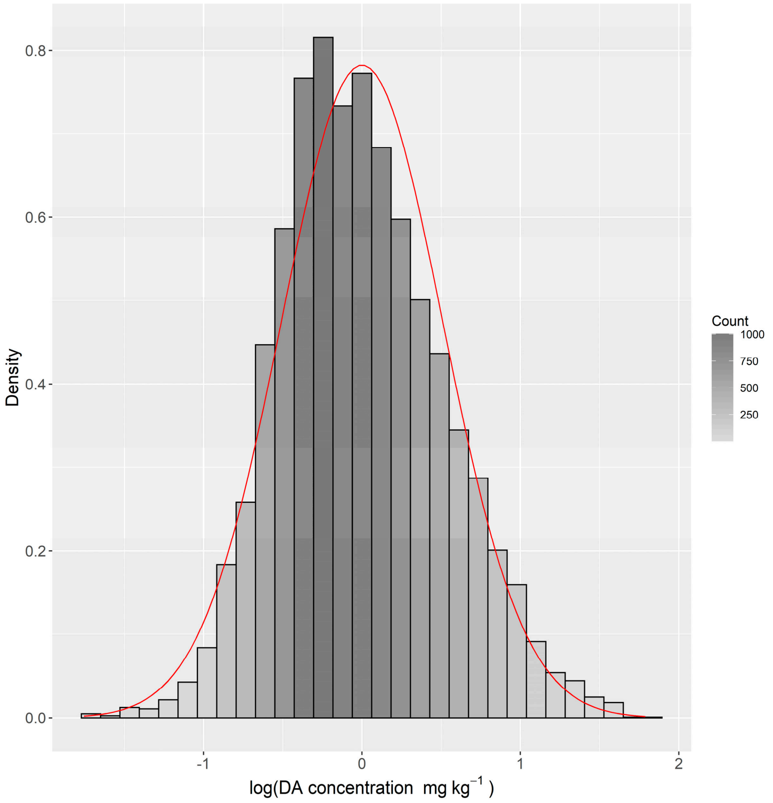

Figure S1. Frequency distribution of the domoic acid concentrations (residuals of the two-way ANOVA Estuary-Species applied to the logarithmically transformed data) in the main bivalve species during the sampling period, and its fitted normal distribution.

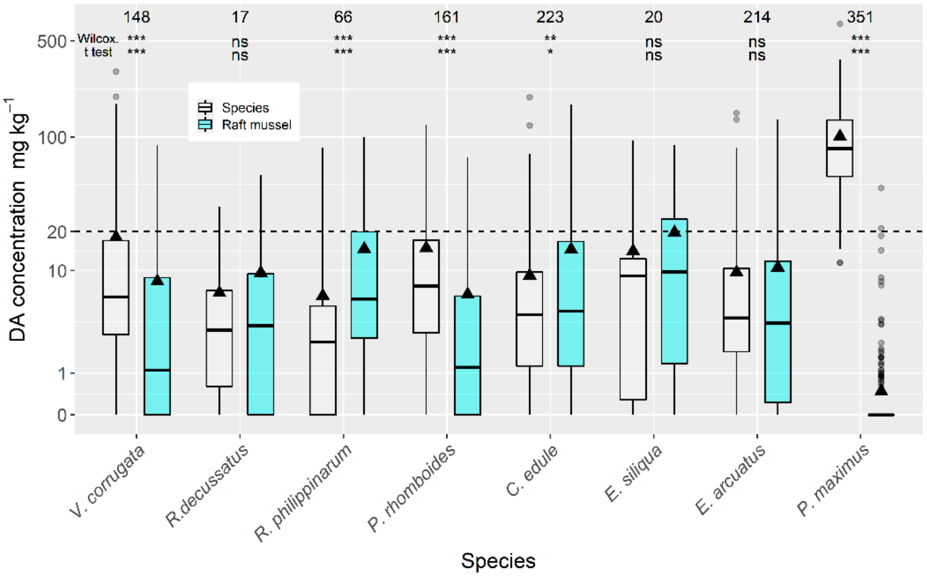

Figure S2. Comparison of DA concentration in raft mussel and other bivalve species, using data (maximum) collected from the same week and the same area. Mean, 25, 50 and 75% quantiles, range excluding outliers and outliers, are represented by triangles, horizontal lines of the box, extremes of the vertical lines from the box and dots, respectively. The dashed line represents the regulatory threshold. The numbers at the top are the number of observations, and the symbols at the bottom are the significance levels estimated by paired Wilcoxon and *t* tests.

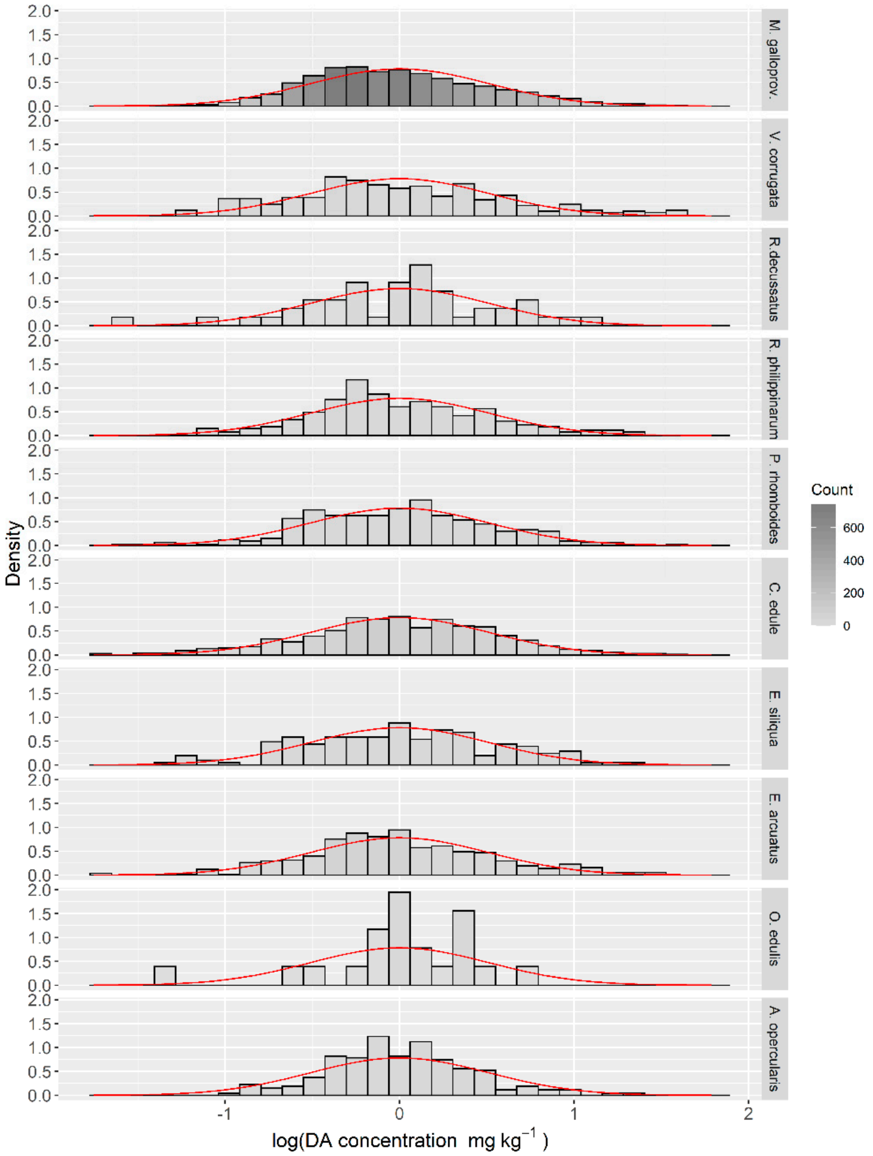

Figure S3. Frequency distribution of the domoic acid concentrations (residuals of the two- way ANOVA Ría-Species applied to the logarithmically transformed data) of each studied species during the sampling period, and their corresponding fitted normal distributions.

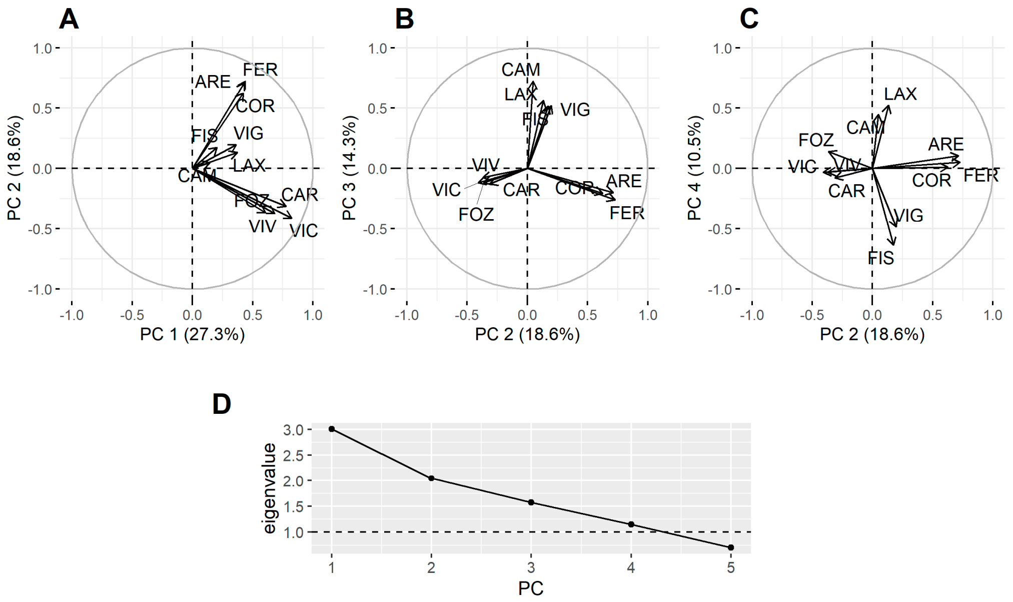

Figure S4. Loadings of the four principal components and their corresponding eigenvalues after excluding king scallops and raft mussels from the analysis. All data but those corresponding to the king scallop and raft mussel were included.

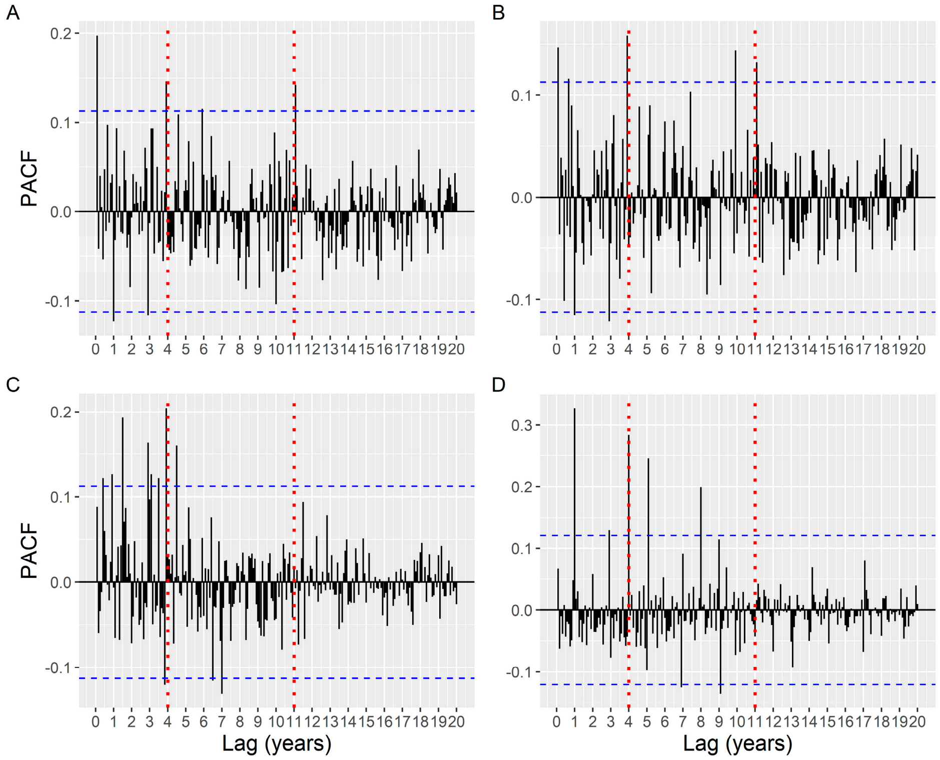

Figure S5. Partial autocorrelation plots corresponding to all Rías **(A)**, and to the southern **(B)**, middle **(C)**, and northern ones **(D)**. Horizontal dashed lines are the 95 % confidence limits and the dotted vertical ones indicate the 4 and 11 year periodicities.

Table S1. Percentage of the total number of samples analyzed corresponding the each bivalve species.

Bivalve	% Of Samples
M. galloprovincialis Raft	74.6506049
M. galloprovincialis Wild	12.9653842
V. corrugata	1.3391894
R.decussatus	0.2005611
R. philippinarum	1.2363701
P. rhomboides	0.6829231
C. edule	5.0381447
E. siliqua	0.6714987
E. arcuatus	1.4991305
M. gigas	0.0342731
O. edulis	0.0939337
P. maximus	1.1183183
A. opercularis	0.4696683
